# COVID-19 in children with haematological malignancies

**DOI:** 10.1136/archdischild-2021-322062

**Published:** 2021-07-22

**Authors:** Gerard Cathal Millen, Roland Arnold, Jean-Baptiste Cazier, Helen Curley, Richard Feltbower, Ashley Gamble, Adam Glaser, Richard G Grundy, Laura Kirton, Lennard Y W Lee, Martin G McCabe, Claire Palles, Bob Phillips, Charles A Stiller, Csilla Varnai, Pamela Kearns

**Affiliations:** 1 Cancer Research UK Clinical Trials Unit, University of Birmingham Institute of Cancer and Genomic Sciences, Birmingham, UK; 2 Department of Paediatric Oncology, Birmingham Women's and Children's NHS Foundation Trust, Birmingham, UK; 3 Institute of Cancer and Genomic Sciences, University of Birmingham, Birmingham, UK; 4 Centre for Computational Biology, University of Birmingham, Birmingham, UK; 5 Leeds Institute for Data Analytics (LIDA), University of Leeds School of Medicine, Leeds, West Yorkshire, UK; 6 CCLG Executive, Children's Cancer and Leukaemia Group, Leicester, UK; 7 Leeds Institute of Medical Research, University of Leeds, Leeds, West Yorkshire, UK; 8 School of Medicine, University of Nottingham Children's Brain Tumour Research Centre, Nottingham, UK; 9 Department of Oncology, University of Oxford, Oxford, Oxfordshire, UK; 10 Division of Cancer Sciences, The University of Manchester, Manchester, UK; 11 National Cancer Registration and Analysis Service, Public Health England, London, UK; 12 Centre for Reviews and Dissemination, University of York Alcuin College, York, UK; 13 Paediatric Oncology, Leeds Children's Hospital, Leeds, West Yorkshire, UK

**Keywords:** COVID-19, epidemiology, data collection

## Abstract

**Background:**

Children with cancer are not at increased risk of severe SARS-CoV-2 infection; however, adults with haematological malignancies have increased risk of severe infections compared with non-haematological malignancies.

**Methods:**

We compared patients with haematological and non-haematological malignancies enrolled in the UK Paediatric Coronavirus Cancer Monitoring Project between 12 March 2020 and 16 February 2021. Children who received stem cell transplantation were excluded.

**Results:**

Only 2/62 patients with haematological malignancy had severe/critical infections, with an OR of 0.5 for patients with haematological compared with non-haematological malignancies.

**Interpretation:**

Children with haematological malignancies are at no greater risk of severe SARS-CoV-2 infection than those with non-haematological malignancies.

## Abstract (precis)

Children have a very low risk of developing severe SARS-CoV-2 infections. Early in the pandemic it became clear from the experiences in China, Italy and Spain that this was also true for children with cancer. Not so for adult patients particularly those with haematological malignancies who were considered to be 'extremely vulnerable'. The recommendation however, from the UK's national Childhood Cancer and Leukaemia group (CCLG) in 2020 was that, other than particularly high risk patients (those with multiply relapsed disease or undergoing bone marrow transplant or CAR-T therapy) children with cancer, including those with haematological malignancies, need not be considered extremely vulnerable. This clearly written, concise paper by Millen et al compares patients with haematological and non-haematological malignancies enrolled on the UK Paediatric Coronavirus Cancer Monitoring Project from March 2020 until Feb 2021 and supports that recommendation.

**What is already known?**

- Children with cancer are not at increased risk of severe infection with SARS-CoV-2 compared with the normal paediatric population. Adults with haematological malignancies are at increased risk of severe forms of SARS-CoV-2 infection and death compared with adults with other malignancies.

**What this study adds?**

- Children with haematological malignancies are not at any increased risk of severe SARS-CoV-2 infection than children with other malignancies. This is clearly different from adult patients and supports the continuation of routine care for these patients.

## Introduction

Data previously published by our group have suggested that children with cancer are at no greater risk of severe infection with the coronavirus SARS-CoV-2 than other children.^1^ The UK Coronavirus Cancer Monitoring Project (UKCCMP) identified that adults with haematological malignancies were more likely to have a severe/critical phenotype than patients with non-haematological malignancies.^2^

Given that approximately 40% of children with cancer are diagnosed with a haematological malignancy, we performed a sub-analysis on our dataset to examine the severity of SARS-CoV-2 in this population.

## Methods

The UK Paediatric Coronavirus Cancer Monitoring Project (UKPCCMP) is a national database of children less than 16 with an underlying diagnosis of malignancy attending hospital with evidence of COVID-19 infection.^1^ The database was designed as a public health surveillance registry to support rapid clinical decision-making, in accordance with the UK Policy Framework for Health and Social Care Research, the UK National Research Ethics Service and the UK Governance Arrangement for Research Ethic Committees. Research Ethics Committee approval was not required, as confirmed by the Health Research Authority. Reporting is undertaken by named professionals in all 20 UK paediatric oncology Principal Treatment Centres.

Patients were eligible for inclusion if they had a positive SARS-CoV-2 reverse transcriptase polymerase chain reaction (RT-PCR) test between 12 March 2020 and 16 February 2021 and had an underlying diagnosis of any subtype of haematological malignancy including (but not limited to) all subtypes of leukaemia and lymphoma as well as myelodysplastic syndromes. Patients with other malignancies in the UKPCCMP cohort were used as a comparator. The cohort included both symptomatic and asymptomatic patients screened prior to elective admission to hospital.

Patients who had undergone an allogeneic stem cell transplant were excluded from the database as their information is being reported separately by the European Society for Blood and Marrow Transplantation (https://www.ebmt.org/covid-19-and-bmt).

An OR was estimated with 95% CIs to describe the risk of developing a severe/critical phenotype of COVID-19 in the haematological malignancy subgroup compared with the non-haematological malignancy subgroup. The latter group included patients with various CNS tumours, sarcomas, neuroblastomas and other, rarer tumours.

## Results

Up to 16 February 2021, a total of 110 patients were registered; 62 (56%) had a haematological malignancy. Table 1 shows the demographics and severity of COVID-19 infection in both groups.

**Table 1:** Demographics and severity of SARS-CoV-2 infection in the UKPCCMP cohort

		UKPCCMP haematological cohort	UKPCCMP non-haematological cohort
Sex			
	Male	35 (56%)	34 (71%)
	Female	27 (44%)	14 (29%)
Median age (range)		7 years, 7 months (4 months to 15 years, 9 months)	3 years, 10 months (1 month to 15 years, 9 months)
Ethnicity			
	White	37 (59.7%)	23 (47.9%)
	Black	3 (4.8%)	4 (8.3%)
	Asian	13 (21%)	8 (16.7%)
	Other	3 (4.8%)	4 (8.3%)
	Undisclosed	6 (9.7%)	9 (18.8%)
Severity of SARS-CoV-2			
	Asymptomatic	19 (30.6%)	17 (35.4%)
	Mild	39 (62.9%)	27 (56.2%)
	Moderate	2 (3.2%)	1 (2.1%)
	Severe	1 (1.6%)	1 (2.1%)
	Critical	1 (1.6%)	2 (4.2%)

UKPCCMP, UK Paediatric Coronavirus Cancer Monitoring Project.

In total, 2/62 patients (3.2%) with haematological malignancies had severe/critical infections. Both patients had pre-B acute lymphoblastic leukaemia; one was receiving delayed intensification and the other was receiving blinatumumab for non-remission after induction.

Also, 3/48 patients (6.3%) with non-haematological malignancies had severe/critical infections. One had a hepatoblastoma on a background of chronic lung disease. One patient had an osteosarcoma and was receiving postoperative chemotherapy. The final patient had a refractory stage 4 neuroblastoma.

Most patients with a positive SARS-CoV-2 test and haematological malignancy had acute lymphoblastic leukaemia (ALL) (table 2).

**Table 2:** Underlying diagnosis of patients with haematological malignancies in the UKPCCMP cohort

Diagnosis	Subtype	Number (%)
Leukaemia		57 (92)
	Acute lymphoblastic leukaemia	48 (77.4)
	Acute myeloid leukaemia	8 (12.9)
	Acute leukaemia, not otherwise specified	1 (1.6)
Lymphoma		5 (8)
	Hodgkin’s lymphoma	3 (4.8)
	Burkitt’s lymphoma	2 (3.2)

UKPCCMP, UK Paediatric Coronavirus Cancer Monitoring Project.

In the UKPCCMP cohort, we did not identify any increased risk of severe/critical infection in children with haematological compared with non-haematological malignancies. The OR for severe/critical disease in patients with haematological compared with non-haematological disease was 0.50 (95% CI 0.08 to 3.11). Also, 80% of patients in the haematological cohort were receiving their first line of chemotherapy compared with 83% of patients in the non-haematological cohort.

## Discussion

In this updated analysis on our initial review of the severity of COVID-19 in children with cancer in the UK, we focus specifically on comparing patients with haematological and non-haematological malignancies.

Since the beginning of the pandemic, a number of groups including our own have published data on the severity of COVID-19 in children with cancer and on the severity of COVID-19 in patients with different haematological malignancies.^3–5^ This cohort specifically compares the severity of infection between paediatric patients with haematological and non-haematological malignancies.

The adult UKCCMP reported that patients with haematological malignancies have a significantly higher case-fatality rate than patients with non-haematological malignancies (OR 2.25, 95% CI 1.13 to 4.57).^2^ Similar findings have been reported by other groups.^6 7^

In our haematological malignancy cohort, there were no deaths related to COVID-19 and only two patients required intensive care support. Also, 31% of patients were asymptomatic and were identified either through targeted screening prior to a general anaesthetic or were admitted for another reason and incidentally found to be SARS-CoV-2 positive. In the non-haematological cohort, 36% were asymptomatic. Thus, there was no evidence of a higher rate of asymptomatic cases in the haematological cohort despite a higher rate of general anaesthetics, suggesting that the estimated OR of 0.50 for developing severe or critical disease was not due to higher rates of detection by asymptomatic screening. In the adult UKCCMP cohort, 119/227 patients with haematological malignancies (52.4%) had severe or critical infections. They had an increased risk of severe/critical infections compared with those with non-haematological malignancies (OR 1.55, 95% CI 1.16 to 2.09).^2^

Given the OR for severe/critical disease in patients with haematological malignancies compared with non-haematological malignancies was 0.5, there is a possibility that the risk of severe/critical infection may actually be higher in children with non-haematological malignancies, but the small numbers make it difficult to draw any conclusion.

In the UKPCCMP cohort, 63.6% of patients had a mild infection that either did not require admission to hospital or had a short stay with no need for additional therapy such as supplemental oxygen. This figure remains consistent with our previously reported cohort in which 63% of patients had a mild infection, highlighting the stability of the data over time.

Approximately 40% of children diagnosed with cancer across the UK have a haematological malignancy,^8^ but 54% of patients in the UKPCCMP paediatric cohort had a haematological malignancy, 76% of whom had ALL. Treatment for ALL lasts 2–3 years, compared with 6 months of treatment for most other malignancies, resulting in an on-treatment prevalence for ALL that is higher than its incidence. This may partly explain the high proportion of patients with ALL in our cohort. Others have proposed that the relatively greater and more prolonged lymphopenia of patients with haematological malignancies may render them more susceptible to contracting viral infections in general including SARS-CoV-2.^6^

The major strength of our study is that all principal treatment centres caring for children with cancer in the UK were enrolled. While some patients with positive SARS-CoV-2 PCR results may not have been registered on the UKPCCMP database, rigorous and repeated follow-up with participating centres has ensured near complete capture of childhood cancers attending hospital in the reported timeframe.

The major limitation of our study is the relatively small numbers of patients involved. However, the results shown here are remarkably similar to the data we published for all children with cancer last year. A specific limitation with regard to our haematological cohort is our elective omission of patients who had undergone an allogeneic stem cell transplant as they were being captured by another registry study. This decision does limit our ability to generalise our findings to all patients with haematological malignancies, but the conclusion that paediatric patients with cancer are at no greater risk of severe COVID-19 infection remains relevant for those who have not had an allogeneic transplant. Furthermore, less than 1% of patients with haematological malignancies in the adult UKCCMP cohort had allogeneic stem cell transplants (personal communication). This therefore means that, although we excluded patients who were post-allogeneic transplant from our dataset, the two sets are largely comparable.

## Conclusion

Our data suggest that children with haematological malignancies who have not had an allogeneic stem cell transplant are not at greater risk of severe infection with SARS-CoV-2 than other children with cancer. This supports the recommendations that children with haematological malignancies need not be considered extremely vulnerable and should continue with standard of care therapy without modification attendant on the SARS-CoV-2 pandemic.

## Data Availability

Data are available on reasonable request. The database contains de-identified participant data which are anonymised at source prior to upload. Reasonable requests for data sharing will be considered.

## References

[1] Millen GC , Arnold R , Cazier J-B , et al . Severity of COVID-19 in children with cancer: report from the United Kingdom Paediatric Coronavirus Cancer Monitoring Project. Br J Cancer 2021;124:754–9. 10.1038/s41416-020-01181-0 33299130 PMC7884399

[2] Lee LYW , Cazier J-B , Starkey T , et al . COVID-19 prevalence and mortality in patients with cancer and the effect of primary tumour subtype and patient demographics: a prospective cohort study. Lancet Oncol 2020;21:1309–16. 10.1016/S1470-2045(20)30442-3 32853557 PMC7444972

[3] Bisogno G , Provenzi M , Zama D , et al . Clinical characteristics and outcome of severe acute respiratory syndrome coronavirus 2 infection in Italian pediatric oncology patients: a study from the infectious diseases Working group of the Associazione Italiana di Oncologia E Ematologia Pediatrica. J Pediatric Infect Dis Soc 2020;9:530–4. 10.1093/jpids/piaa088 32652521 PMC7454778

[4] Faura A , Rives S , Lassaletta Álvaro , et al . Initial report on Spanish pediatric oncologic, hematologic, and post stem cell transplantation patients during SARS-CoV-2 pandemic. Pediatr Blood Cancer 2020;67:e28557. 10.1002/pbc.28557 32672895 PMC7404582

[5] Ferrari A , Zecca M , Rizzari C , et al . Children with cancer in the time of COVID‐19: an 8‐week report from the six pediatric onco‐hematology centers in Lombardia, Italy. Pediatr Blood Cancer 2020;67. 10.1002/pbc.28410 PMC7267084 32452123

[6] Jee J , Foote MB , Lumish M , et al . Chemotherapy and COVID-19 outcomes in patients with cancer. J Clin Oncol 2020;38:3538–46. 10.1200/JCO.20.01307 32795225 PMC7571792

[7] Kuderer NM , Choueiri TK , Shah DP , et al . Clinical impact of COVID-19 on patients with cancer (CCC19): a cohort study. Lancet 2020;395:1907–18. 10.1016/S0140-6736(20)31187-9 32473681 PMC7255743

[8] CRUK . Children’s cancers incidence statistics, 2015. Available: https://www.cancerresearchuk.org/health-professional/cancer-statistics/childrens-cancers/incidencefiles/686/incidence.html [Accessed 10 Feb 2021].

